# Systematic review and meta-analysis on the effects of chronic peri-adolescent cannabinoid exposure on schizophrenia-like behaviour in rodents

**DOI:** 10.1038/s41380-024-02668-5

**Published:** 2024-08-02

**Authors:** Zhikun Li, Diptendu Mukherjee, Bea Duric, Isabelle Austin-Zimmerman, Giulia Trotta, Edoardo Spinazzola, Diego Quattrone, Robin M. Murray, Marta Di Forti

**Affiliations:** 1 Social, Genetic and Developmental Psychiatry Centre, Institute of Psychiatry, Psychology and Neuroscience, King’s College London, London, SE5 8AF UK; 2 MRC Centre for Neurodevelopmental Disorders, Institute of Psychiatry, Psychology and Neuroscience, King’s College London, London, London, SE1 1UL UK; 3 GKT School of Medical Education, King’s College London, London, SE1 1UL UK; 4 South London and Maudsley NHS Mental Health Foundation Trust, London, UK; 5 Department of Psychosis Studies, Institute of Psychiatry, King’s College London, De Crespigny Park, Denmark Hill, London, SE5 8AF UK; 6 National Institute for Health Research (NIHR) Mental Health Biomedical Research Centre at South London and Maudsley NHS Foundation Trust and King’s College London, London, UK

**Keywords:** Molecular biology, Schizophrenia, Neuroscience

## Abstract

**Background:**

The link between cannabis use and schizophrenia is well-established in epidemiological studies, especially among adolescents with early-onset use. However, this association in rodent models is less clear. This meta-analysis examined the effects of adolescent cannabinoid exposure on distinct schizophrenia-like behaviours in rodents and how experimental variations influence outcomes.

**Methods:**

Following a pre-registered protocol (CRD42022338761), we searched PubMed, Ovid Medline, Embse and APA PsychInfo for English-language original studies until May 2024. We synthesised data from experiments on schizophrenia-like behaviour in rats and mice after repeated peri-pubertal (onset between P23-P45) cannabinoid exposure. Risk of bias was assessed using the SYRCLE’s tool.

**Results:**

We included 359 experiments from 108 articles across 9 behavioural tests. We found meta-analytic evidence supporting that CB1R agonists, both natural and synthetic, elicited broad schizophrenia-like behavioural alterations, including impaired working memory [*g*  = −0.56; (CI: −0.93, −0.18)], novel object recognition [*g* = −0.66; (CI: −0.97, −0.35)], novel object location recognition [*g* = −0.70; (CI: −1.07, −0.33]), social novelty preference [*g* = −0.52; (CI: −0.93, −0.11)], social motivation [*g* = −0.21; (CI: −0.42, −0.00)], pre-pulse inhibition [*g* = −0.43; (CI: −0.76, −0.10)], and sucrose preference [*g* = −0.87; (CI: −1.46, −0.27)]. By contrast, effects on novelty-induced locomotion were negligible. Subgroup analyses revealed similar effects across sexes and species. Substantial variance in the protocols and moderate-to-high heterogeneity in behavioural outcomes were observed. We found CBD may enhance fear memory recall, but data was limited.

**Discussion:**

This is the first meta-analysis to comprehensively assess the link between cannabinoids and schizophrenia-like behaviours in rodents. Our results support epidemiological links between early cannabis use and schizophrenia-like phenotypes, confirming the utility of animal models. Standardising protocols will optimise models to strengthen reproducibility and comparisons, our work provides a framework for refining rodent models to elucidate biological pathways linking cannabis and schizophrenia.

## Introduction

Cannabis is one of the oldest and most widely used psychoactive substances in human history [1]. The latest UN report estimated that the global number of cannabis users reached 209 million in 2020, representing a 23% increase from 2010 [2]. The increasing popularity of cannabis use may be influenced by several factors, such as the legalisation of medicinal and recreational cannabis in some countries, the increased availability and social acceptance, and the perception that cannabis has low health risks [3, 4]. However, epidemiological studies have consistently shown a link between frequent cannabis use and psychosis [5, 6]. Recent evidence showed that daily cannabis users had a three-fold higher risk of developing psychosis than non-users. The risk increases with the use of high-potency cannabis that contains high levels of tetrahydrocannabinol (THC), the main psychoactive ingredient in cannabis [7]. Furthermore, evidence suggested that earlier adolescent cannabis initiation may also confer greater psychosis vulnerability [8]. Despite this knowledge, the biological mechanisms underlying this relationship, and the role of genetics have not been conclusively elucidated [9].

Human studies on cannabis and psychosis face considerable challenges in controlling for genetics, cannabis type, consumption patterns and social contexts. In contrast, rodent models enable controlled examination of cannabis exposure effects on the brain and behaviour. Given the conserved nature of brain circuits between humans and rodents [10, 11], animal models hold promise for probing the pathophysiological mechanisms underlying cannabis effects relevant to humans.

Over the years, a growing body of research has investigated the impact of cannabinoids on schizophrenia-like behaviours in rodents [12–16]. While providing useful preliminary evidence, these studies have substantial variability in factors like cannabinoid type and dosage, timing of exposure, sex, and species of animals used [14]. Additionally, while numerous behavioural aspects have been reported to be impacted by cannabis exposure in rodents, the validity and sensitivity of the individual tests used to model complex schizophrenia-like behaviour in rodents remain unclear.

To address these questions, we conducted a systematic review and meta-analysis of rodent experiments that modelled chronic cannabis use and assessed its link with schizophrenia-like behaviours. We focused on studies that administered cannabinoids during adolescence, a critical neurodevelopmental period with heightened vulnerability to substance impacts [17–21]. Through this meta-analysis, we aimed to 1) summarise existing behavioural data of rodent experiments that modelled adolescent cannabis exposure, 2) compare the impacts of distinct cannabinoids, particularly THC and cannabidiol (CBD), 3) explore the potential moderating factors of sex, species, time lapse between treatment and assessment (short-term vs long-term), and 4) discuss the implications for future research and identify open questions in the field of rodent models of cannabinoids exposure.

## Methods

This systematic review and meta-analysis followed the PRISMA (Preferred Reporting Items for Systematic Reviews and Meta-Analyses) guidelines (Supplementary Appendix 1). The protocol was pre-registered at PROSPERO on 21st June 2022, protocol number: CRD42022338761.

### Search strategy

The literature search was conducted on 5th May 2024 across electronic databases including PubMed, EMBASE, MEDLINE and APA PsycINFO, using the following keywords: 1) cannabis, 2) animal, and 3) adolescence. (Full search terms in Supplementary appendix 2). We included peer-reviewed original studies written in English.

### Study screening and eligibility criteria

The titles and abstracts of 2806 articles retrieved from the preliminary search were screened and cross-checked by two independent reviewers (ZL and BD) and discrepancies were resolved through discussion with a third researcher (DM). Subsequently, the two reviewers evaluated the full texts of the remaining articles using specific inclusion criteria adapted from the PICO method [22] (Additional details in Supplementary Appendix 3). We pre-specified 12 behavioural tests [Table 1] relevant to the three core domains of schizophrenia symptoms: positive symptoms, negative symptoms and cognitive impairments [23–26]. We pooled data from each behavioural test into separate meta-analyses.

**Table 1:** Behavioural tests and primary outcome measures for data extraction.

	Relevant schizophrenia symptoms												
	Positive symptoms		Social withdrawal		Working memory		Short-term memory		Sensori-motor gating	Associative memory	Spatial memory	Cognitive flexibility	Anhedonia
Test	Locomotion in open field		Social motivation	Social novelty preference	Y maze	T maze	Novel object recognition	Novel object location	Pre-pulse inhibition	Fear conditioning	Morris water maze	Attentional set-shifting	Sucrose preference
	novelty induced	Psycho- stimulant-induced											
Primary outcome measure	Total distance travelled in the open field		Social motivation index	Social preference index	%Correct alternations		Discrimination Index		%PPI	%Time freezing in recall trial	Time to reach hidden platform	Number of trials to reach criterion in shift trial	Sucrose preference index

### Data extraction strategy and quality assessment

Two reviewers (ZL and BD) independently extracted data using a standardised data extraction form (Supplementary Data Collection Sheet). Importantly, we regarded a comparison between a control and a cannabinoid treatment group as a single experiment, and an effect size was calculated for each such comparison/experiment. Therefore, if an article included multiple control-treatment comparisons, each comparison was treated as one separate experiment, and multiple effect estimate data from that article were calculated and pooled.

Quality assessment was performed following the SYRCLE’s risk of bias tool for animal studies [27], which evaluated each article for their risk of bias across 10 items, assigning a rating of high risk, low risk, or unclear for each item.

### Statistical analysis

Analyses were performed in R, using packages *meta*, *metafor* and *RevMan*. From each experiment, we calculated the effect sizes as Hedge’s *g*. The inverse variance-weighted random effects model with Knapp-Hartung adjustment was used to calculate overall effect sizes. The Restricted Maximum-Likelihood (REML) estimator was used for τ^2^.

The direction of effect sizes reflects the numerical change of effect in the experiment group compared to the control group. Data are presented as Hedge’s *g* ± 95% confidence intervals. Results were regarded as significant when the confidence interval entirely excluded zero and corresponded to a *p* value lower than 0.05 in Cochran’s Q test. A summary effect was considered valid only when at least 4 individual effect estimates could be pooled.

Heterogeneity was assessed using the *I*^*2*^ statistics [28]. We regarded an *I*^*2*^ of 0–40%: might not be important; 30–60%: may represent moderate heterogeneity; 50–90%: may represent substantial heterogeneity; 75–100%: considerable heterogeneity [29].

The pre-specified list of subgroup analyses included assessments for the effect of a) species, b) sex, c) substance, d) time lapse of behavioural assessment post treatment. Cochran’s Q statistics was used to assess between-subgroup differences. We regarded a subgroup result as valid only when there was a minimum number of 4 experiments in each subgroup.

Publication bias was inspected qualitatively by assessing the asymmetry of funnel plots and quantitatively by the Egger’s test [30]. Sensitivity analyses were conducted to evaluate the robustness of the overall effect estimates when excluding studies a) with high-risk level of bias and b) reported alternative outcome measures.

## Results

**Fig. 1:**
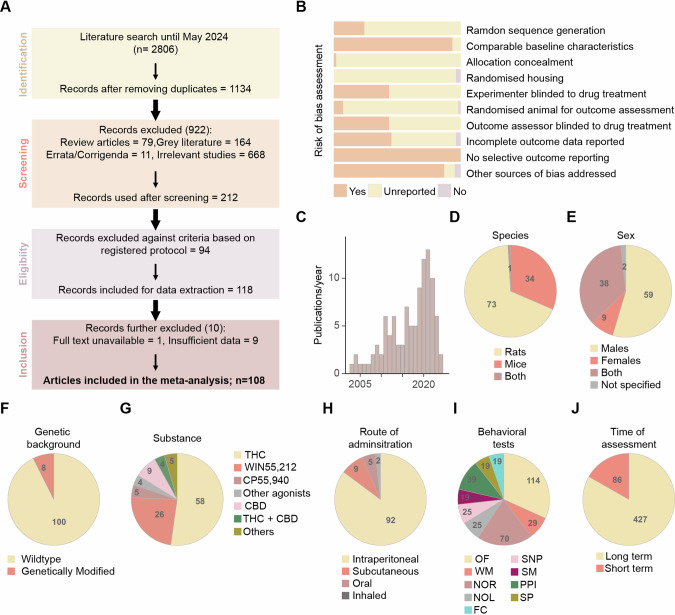
Article screening and general characteristics of the studies included. **A** Flowchart of the screening strategy for the articles returned from electronic databases and the number of studies excluded at each step. **B** Cumulative bar charts displaying risk of bias assessment across 10 items specified in SYRCLE’s assessment tool. **C** Histogram showing the trend in articles published every year between 2003 and 2024 included in the meta-analysis. Pie charts depicting the proportion and number of studies categorised by species (**D**), sex (**E**), genetic background (**F**), substance administered (**G**), route of administration (**H**), the number of behavioural tests reported (**I**) and timing of behavioural tests (short term: 24 h to 10 d after final dose; long term: >10 d after final dose) (**J**). OF novelty-induced locomotion in an open field, WM working memory, NOR novel object recognition, NOL novel object location, SNP social novelty preference, PPI prepulse inhibition, FC fear conditioning, SM social motivation, SP sucrose preference.

A total of 2806 records were identified through database search, amongst which 118 studies matched our inclusion criteria. 10 articles were subsequently excluded for insufficient data, resulting in *n* = 108 studies published between 2003 and 2024 being used for quantitative data synthesis [Fig. 1A, C; Supplementary Data Sheet].

Risk of bias assessment [Fig. 1B] revealed all included articles pre-specified outcome measures. Most articles adequately controlled and reported baseline animal characteristics (93.5%) and provided sources of funding/conflict of interest statements (87%). However, few articles disclosed sufficient details on randomisation procedures and blinding methods as specified in the SYRCLE assessment tool. Therefore, the majority of studies were labelled “unclear risks” for these domains.

To determine the diversity of experimental paradigms applied, we categorised studies according to the genetic background, species, sex, substance administered, route of drug administration, behavioural measures, and the timing of behavioural assessment. These data are presented as pie charts [Fig. 1D–K]. We found that most studies (*n* = 73) used rats as the model organism, while *n* = 34 studies used mice, and one study characterised both species [Fig. 1D]. Over half of the studies included only one sex, with male animals (*n* = 59) being preferred over females (*n* = 9) [Fig. 1E].

While the majority of the studies used wildtype animals, 8 studies used genetically modified mice [Fig. 1F, a descriptive summary of gene-environment interaction findings in Supplementary appendix 4]. Regarding substances, the effects of THC (*n* = 58) have been extensively profiled, followed by the synthetic full CB1R agonists WIN-55212-2 (*n* = 26) and CP55,940 (*n* = 5) [Fig. 1G]. We also observed substantial variance in the tested THC doses (ranging from 0.2 mg/kg to 15 mg/kg), plus 77 experiments adopting an escalating dose paradigm (e.g., 2.5–5–10 mg/kg). Furthermore, non-contingent methods of drug delivery [31] such as intraperitoneal (*n* = 92) or subcutaneous injection (*n* = 9) were more common than voluntary self-administration [Fig. 1H].

Corresponding to the schizophrenia symptoms in humans, we specified a list of 12 behavioural tasks. From all the experiments (K = 359) synthesised from the 108 articles, we registered 9 behavioural tests that were reported in more than 4 experiments. Morris water maze (K = 10), attentional set-shifting (K = 3), and psychostimulant-induced hyperactivity in an open field (K = 5) were excluded due to data inaccessibility or lack of suitable outcome measure. Amongst the 9 behavioural tests, novelty-induced locomotor activity in an open field test was most examined (K = 114), followed by novel-object recognition (K = 70) [Fig. 1I]. Within our specified range of administration onset around puberty (P21-56), most studies began treatment in week 5 (P28-P34). Finally, while a small portion of the experiments (K = 86) examined the short-term effects of adoslecent cannabinoid treatments, most studies performed experiments (K = 427) after an abstinent period of more than ten days exclusively or repeatedly after short-term experiments [Fig. 1J].

### Exposure to CB1R agonists and schizophrenia-like behaviours in rodents

Exposure to CB1R agonists was associated with a detrimental effect on working memory tasks [*g*  = −0.56; 95% CI = (−0.93; −0.18), *p* = 0.005; *n* = 28] and short-term memory tests including novel object recognition [*g* = −0.66; 95% CI = (−0.97; −0.35), *p*  < 0.0001, *n* = 63], novel object location [*g* = −0.70; 95% CI = (−1.07; −0.33), *p* < 0.005, *n* = 25], social novelty preference [*g* = −0.52; 95% CI = (−0.93; −0.11), *p*  < 0.05, *n* = 25], as well as sensorimotor gating assessed through pre-pulse inhibition [*g* = −0.43; 95% CI = (0.76; −0.1), *p* < 0.05, *n* = 33]. Rodents exposed to CB1R agonists also displayed behaviour related to negative symptoms, such as reduced social motivation [*g* = −0.21; 95% CI = (−0.42; −0.005), *p*  <  0.05, *n* = 18]. There was also reduced sucrose preference behaviour [*g* = −0.87; 95% CI = (−1.46; −0.27), *p* < 0.05, *n* = 14] and a trend towards impaired 24 h fear memory recall [*g* = −0.45; 95% CI = (−0.91; 0.00), *p* = 0.051, *n* = 11]. Notably, novelty-induced locomotion in an open field was the only behaviour displaying no significant change following adolescent CB1R agonists treatment [*g* = −0.1; 95% CI = (−0.24; 0.04), *p* = 0.15, *n* = 99].

### Comparing behavioural effects of THC and synthetic CB1R agonists

**Fig. 2:**
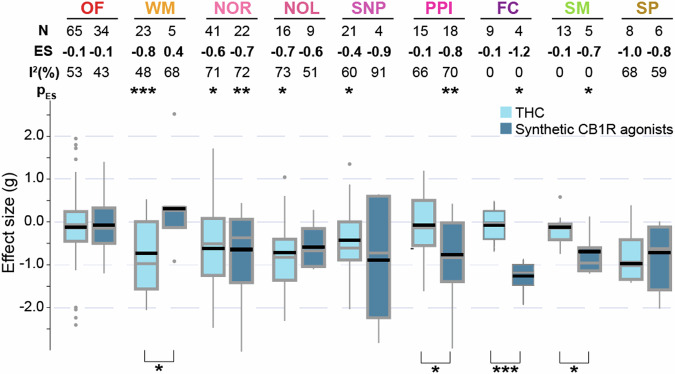
THC and synthetic CB1R agonists are associated with similar schizophrenia-like behavioural modification. Boxplots displaying the distribution of effect estimates (Hedge’s *g*) of THC (light blue) and synthetic CB1R agonists (dark blue) for different behavioural tests; Number of experiments included (N), pooled effect size (ES) and corresponding significance levels are listed on top of each corresponding boxplot. Significant subgroup differences between THC and SC estimated by Cochran’s Q statistics are denoted by asterisks below the boxplots. *p < 0.05, **p < 0.005, ***p < 0.0005. Black horizontal lines in the boxplots indicate pooled effect sizes (inverse variance weighted, mixed effect model), grey horizontal lines indicate medians. Outliers are indicated by grey circles. OF novelty-induced locomotion in an open field, WM working memory, NOR novel object recognition, NOL novel object location, SNP social novelty preference, PPI prepulse inhibition, FC fear conditioning, SM social motivation, SP sucrose preference.

Synthetic Cannabinoids (SCs) encompass a broad class of artificial compounds that mimic the effects of phytocannabinoids such as THC, but often at multiple times higher potency and binding affinity [32]. In our meta-analysis, the SC agonists identified included WIN55,212-2, CP55,940, AB-PINACA, AB-FUBINACA, 5-MDMB-PICA, HU-210 and JWH-018. In a subgroup analysis, we compared behavioural impacts of these SCs versus THC. Overall, THC and SCs produced similar be effects [Fig. 2]. The differences, comparing THC and SCs, were non-significant for for novelty-induced locomotion in an open field (*Q* = 0.11, *p* = 0.74), novel object recognition (*Q* = 0.12, *p* = 0.73), novel object location (*Q* = 0.15, *p* = 0.70), social novelty preference (*Q* = 0.27, *p* = 0.60) and sucrose preference (*Q* = 0.16, *p* = 0.70). However, SCs led to greater impairment than THC social motivation (*Q* = 6.12, *p* = 0.01), pre-pulse inhibition (*Q* = 5.40, *p* < 0.05) and fear conditioning (*Q* = 18.92, *p* < 0.0001). We also observed a significant sub-group effect in working memory tests (*Q* = 4.28, *p* = 0.03), albiet this is likely due to the small sample size and an outlier in the SC group.

### Exposure to CBD and behavioural modulations

**Fig. 3:**
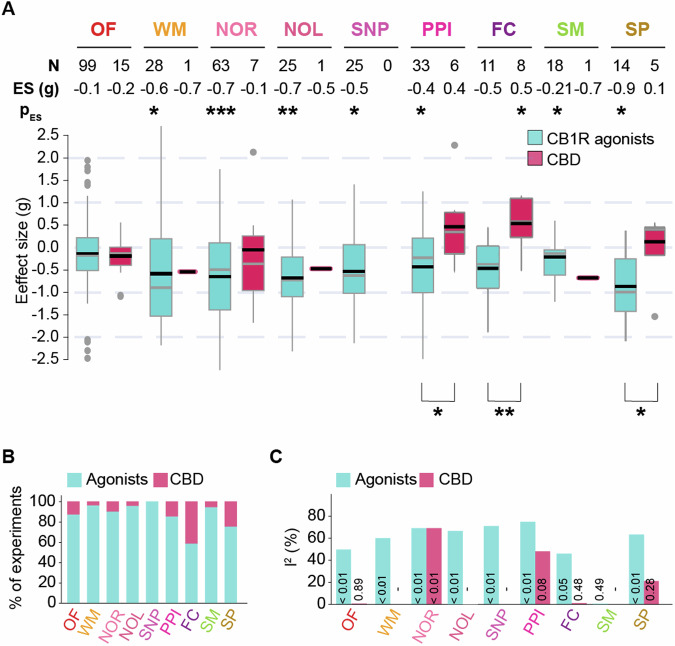
Chronic adolescent exposure to CB1R agonists and CBD differently modulates schizophrenia-like behaviour. **A** Boxplots displaying the distribution of effect sizes (Hedge’s *g*) of CB1R agonists (blue) and CBD (red) across nine behavioural tests; Number of experiments included (N), values of pooled effect sizes (ES) and corresponding significance levels (p_ES_) are listed on top of each corresponding boxplot. Cochran’s Q statistics to compare effect sizes between drug interventions are denoted by asterisks below the boxplots. *p < 0.05, **p < 0.005, ***p < 0.0005. Black horizontal lines in the boxplots indicate pooled effect sizes (inverse variance weighted, random effect model), grey horizontal lines indicate medians. Outliers are indicated by grey dots. **B** Percentage of experiments analysing CB1R agonists (blue) and CBD (red) in each behavioural test. **C** Bar charts displaying heterogeneity of each analysis represented by *I*^*2*^ statistic and associated *p* values shown within each bar. OF novelty-induced locomotion in an open field, WM working memory, NOR novel object recognition, NOL novel object location, SNP social novelty preference, PPI prepulse inhibition, FC fear conditioning, SM social motivation, SP sucrose preference. Forest plots for each behavioural test are available in Supplementary Figs. S3–16.

In contrast to CB1R agonists, we found few studies assessing CBD’s impact that matched our inclusion parameters [Figs. 1G and 3B]. Therefore, we analysed the effect sizes for a subset of behavioural parameters with at least 4 effect estimates pooled [Fig. 3A]. We observed significant effects of CBD in enhancing fear memory retrieval [*g* = 0.53; 95% CI = (0.04; 1.02), *p* < 0.05, *n* = 8]. No significant effect was observed for novelty-induced locomotion in the open field test [*g* = −0.18; 95% CI = ( − 0.42; 0.06), *p* = 0.89, *n* = 15], novel object recognition [*g* = −0.05; 95% CI = ( − 1.18; 1.07), *p* = 0.91, *n* = 7], pre-pulse inhibition [*g* = 0.40; 95% CI = ( − 0.60; 1.41), *p* = 0.34, *n* = 6] and sucrose preference tests [*g* = 0.10; 95% CI = ( − 0.75; 0.95), *p* = 0.77, *n* = 5] with CBD.

Subsequently, comparing the effects of CB1R agonists and CBD, we found a significant difference for pre-pulse inhibition (*Q* = 3.86, *p* < 0.05), sucrose preference (*Q* = 5.50, *p* < 0.05) and fear memory retrieval (*Q* = 11.32, *p* < 0.005). In each of these tests, CBD improved the performance, whereas CB1R agonists worsened it. No difference was detected in the novel object recognition test (*Q* = 1.56, *p* = 0.21) or novelty-induced locomotion in an open field (*Q* = 0.37, *p* = 0.5).

### Heterogeneity, publication bias and sensitivity analysis

We identified moderate to high levels of heterogeneity for most behavioural tests, with *I*^*2*^ statistics ranging between 0 and 73% [Fig. 3C]. To identify potential sources of heterogeneity, statistical outlier analyses were conducted. Outliers were defined as studies in which the 95% confidence interval of the effect size did not overlap with the confidence interval of the pooled effect. Statistical outliers were detected in most behavioural tests, accounting for a portion of the heterogeneity identified for each of the tests (Supplementary Appendix 5).

**Fig. 4:**
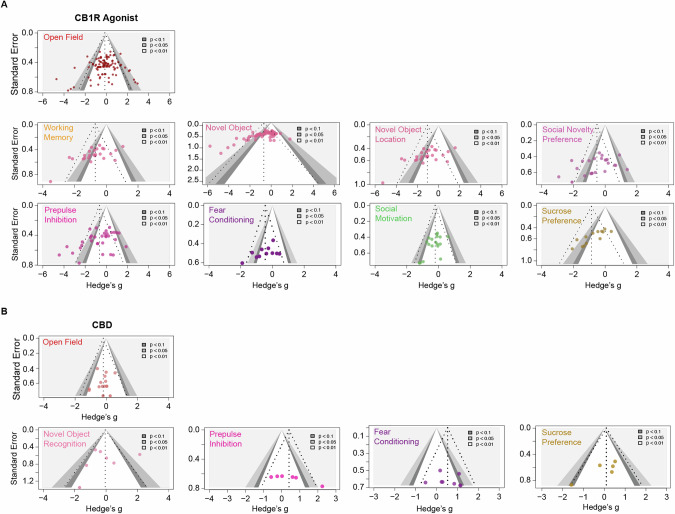
Contour-enhanced funnel plots for assessing potential publication bias. In each plot, effect sizes (Hedge’s *g*) are plotted on the x-axis and standard errors on an inverted y-axis. Each coloured dot on the plot represents a single effect size data point. The dashed lines form an idealised funnel shape that indicates the expected distribution of studies. The vertical line in the middle of the funnel represents the average effect size. The contours from light to dark grey signify the significance levels of the data points, with darker shades epresenting higher significance levels (*p* < 0.01, *p* < 0.05, and *p* < 0.1). **A **Funnel plots displaying the distribution of individual effect estimates from experiments assessing CB1R agonists effects across nine behavioural tests. **B **Funnel plots displaying the distribution of individual effect estimates from experiments assessing CBD effects across five behavioural tests.

We found significant asymmetry in 5 out of 14 funnel plots (4 out of 9 behavioural outcomes measured for CB1R agonist and 1 out of 5 for CBD), indicating publication bias [Fig. 4]. These include novel object recognition [intercept = −3.859, 95% CI = (−5.17; −2.75), *p* < 0.001], novel object location [intercept = −4.976; 95% CI = (−7.99; −1.96), *p* < 0.05], social motivation tests [intercept = −2.401, 95% CI = (−4.3; −0.50), *p* < 0.05], and sucrose preference test for CB1R agonists [intercept = −5.986, 95% CI = (−7.74; −4.23), *p* < 0.01] and pre-pulse inhibition for CBD [intercept = 15.371, 95% CI = (6.44; 24.31), *p* < 0.05], indicating the existence of publication bias.

Furthermore, we performed a sensitivity analysis to assess whether the inclusion of alternative outcome measures would significantly modify the overall effects. Among the 9 behavioural tests, only open field tests assessing novelty-induced locomotion included alternative outcome measures (e.g., beam breaks, number of floor squares entered) in addition to the pre-specified primary measure (total distance travelled) for locomotor activity. The analysis result showed no significant change in pooled effect size before and after removing alternative measures. [ES_total_ = −0.12, 95% CI = (−0.24; 0.28); ES_primary_ = −0.16, 95% CI = (−0.33; 0.01)]. Based on the risk of bias assessment results, we performed sensitivity analyses by comparing effect sizes before and after removing data points with unclear or high risk. We focused on assessment items including random sequence generation, blinding to experimenter, blinding to outcome assessor and addressing incomplete outcome data. Our results sugggest that studies of high and unknown risk had limited impact on the overall effects (see Supplementary Appendix 6 for summary effect sizes including only low risk experiments).

### Behavioural outcomes of chronic adolescent CB1R agonist exposure across rodent species and sexes

To further explore how variations in experiment design might impact behavioural outcomes and contribute to between-study heterogeneities, we performed a series of subgroup analyses. All subgroup analyses were performed with data obtained from studies on CB1R agonists exposure, as CBD studies were sparse.

**Fig. 5:**
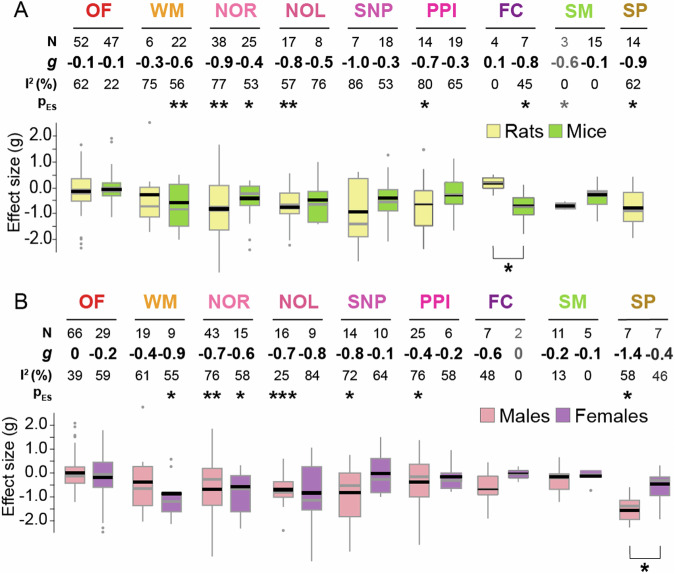
Subgroup analyses revealed similar effects of CB1R agonists across sexes and species. Boxplots displaying the distribution of effect sizes (Hedge’s g) for CB1R agonists in each behavioural test sub-grouped by **A**. species and **B**. sex. Number of experiments (N), pooled effect size values (ES), corresponding *I*^*2*^statistics and *P* values are shown on the top of each corresponding boxplot. Black horizontal lines in the boxplots indicate pooled subgroup effect sizes (inverse variance weighted, mixed effect model), grey horizontal lines indicate medians. Outliers are indicated by grey dots. Significant between-subgroup differences are denoted by asterisks below the boxplots. Comparing the effect sizes between rats and mice: OF novelty-induced locomotion in an open field: *Q* = 0.13, *p* = 0.72; WM Working memory: *Q* = 0.19, *p* = 0.66; NOR Novel object recognition: *Q* = 2.14, *p* = 0.14; NOL Novel object location: *Q* = 0.69, *p* = 0.40; SNP Social novelty preference: *Q* = 1.17, *p* = 0.28; PPI Prepulse inhibition: *Q* = 1.36, *p* = 0.24; FC Fear conditioning: *Q* = 7.25, *p* < 0.05; SM Social motivation, SP sucrose preference. Comparing the effect sizes between males and females: OF- *Q* = 1.00; *p* = 0.31, WM- *Q* = 1.45; *p* = 0.22, NOR- *Q* = 0.12; *p* = 0.73, NOL- *Q* = 0.03; *p* = 0.87, SNP- social novelty preference- *Q* = 3.33; *p* = 0.07, PPI- *Q* = 0.75; *p* = 0.39, SM- *Q* = 0.26, *p* = 0.61; SP- *Q* = 4.15; *p* = 0.04. *p < 0.05, **p < 0.005, ***p < 0.0005.

We first compared the subgroup effect sizes for mice and rats [Fig. 5A]. Significant between-species difference was found only in fear conditioning recall (*Q* = 7.25, *p* < 0.05), albeit all experiments in the rat subgroup came from the same article, which bear the risk of being skewed by the same unidentified confounds. Heterogeneity remained moderate to high in the subgroups, indicating that species was not a significant moderator in our meta-analysis.

Figure 5B displays the subgroup effect sizes for each behavioural test by sex. Notably, 8 out of 9 behavioural tests revealed no significant differences between subgroups. Furthermore, only the female subgroup for social novelty preference showed a substantial reduction in heterogeneity (*I*^*2*^ = 30%) relative to the overall effect, indicating that sex is not a strong moderator for most of the behavioural outcomes.

### Comparing short-term and long-term effects of CB1R agonist exposure

To distinguish the short-term and protracted effects of CB1R agonists, we stratified the data based on the timing of the behavioural tests. Specifically, we categorised tests performed between 24 h to 10 days after the final dose as short-term, and those conducted after an abstinence period of more than 10 days as long-term. As shown in Fig. 1K, most studies assessed behaviour only in the long term. Consequently, we conducted subgroup analyses only for novelty-induced locomotion, novel object recognition, and prepulse inhibition, which each contained more than four short-term outcome data points. Substantial between-subgroup difference was found for novelty-induced locomotion (*Q* = 4.89, *p* < 0.05), where CB1R agonists administration was linked with significantly reduced locomotion in the short-term [*g* = −0.50, 95% CI = (−0.86; −0.13), *n* = 19, *p* < 0.005, *I*^*2*^ = 50%] but not in the long-term [*g* = −0.08, 95% CI = (−0.22; 0.07), *n* = 88, *p* = 0.32, *I*^*2*^ = 47%]. By contrast, there was a significant long-term effect in the prepulse inhibtion test [*g* = −0.50, 95% CI = (−0.89; −0.12), *n* = 28, *p* < 0.05, *I*^*2*^ = 76%], but not in the short term [*g* = −0.15, 95% CI = (−0.63; 0.33), *n* = 6, *p* = 0.51, *I*^*2*^ = 21%]. Similarly, performance in novel object recognition was shown significantly impaired in the long-term [*g* = −0.71, 95% CI = (−1.06; −0.36), *n* = 58, *p* < 0.005, *I*^*2*^ = 73%] but not in the short-term [*g* = −0.89, 95% CI = (−1.92; 0.14), *n* = 8, *p* = 0.08, *I*^*2*^ = 73%]. However, the subgroup comparisons of short-term and long-term effects did not reach statistical significance for novel object recognition (*Q* = 0.14, *p* = 0.70) or prepulse inhibition tests (*Q* = 1.77, *p* = 0.18).

## Discussion

Previous meta-analyses have synthesised preclinical evidence for cannabinoids effects on rodent behaviour related to nociception [33], sleep [34] anxiety and depression [35, 36], and some narrative systematic reviews have addressed aspects of schizophrenia-related behaviour such as social behaviour [37] and cognitive function [38]. However, to our knowledge, this is the first systematic review to meta-analyse results from a comprehensive battery of tests for schizophrenia-like behaviour, focusing on adolescent cannabinoids exposure.

Our meta-analysis revealed a robust association between adolescent exposure to natural and synthetic CB1R agonists and impaired schizophrenia-related behavioural phenotypes in rodents. We report that exposure to CB1R agonists is associated with prominent cognitive deficits and pronounced behaviour changes similar to negative symptoms of schizophrenia. Notably, these effects were persistent even after long-term abstinence. This suggests that adolescent exposure to CB1R agonists may cause lasting disruptions to the brain and impaired behaviour that extends into adulthood in rodents. Our results are consistent with the conclusion from existing reviews on rodent literature [15, 39, 40].

### Locomotor hyperactivity as a proxy for positive symptoms of schizophrenia

Novelty-induced hyperactivity in an open field is often considered a proxy for positive symptoms of schizophrenia [41, 42] due to the well-established connection between dopamine and movement control [43–46]. Indeed, enhanced striatal and subcortical dopaminergic activity has been reported in schizophrenia patients [47–50] and rodents [51, 52]. Although dopaminergic dysfunction is not the only pathophysiological mechanism affected, it is proposed to be a final common pathway where multiple effector pathways converge [53, 54] Therefore, modelling a behaviour that is susceptible to dopamine-dependent changes provides construct validity to this paradigm.

However, we found that adolescent exposure to CB1R agonists did not have a significant overall effect on novelty-induced locomotor activity, and this was overall not modified by sex or species. In a subgroup analysis separating short-term versus long-term effects, we found a significant locomotor suppressing effect when the test was performed more recently to treatment cessation. This could be due to some residual effects of the acute drug-induced hypoactivity commonly observed immediately after drug administration, which subsided during abstinence.

However, the lack of evidence of a long-term effect on locomotion does not necessarily negate the hypothesis that CB1R activation modifies dopaminergic signalling [51, 55, 56]. In fact, chronic long-term exposure to CB1R agonists could have a paradoxical effect on dopamine. Similar to other drug dependence models such as with psychostimulants or opioids [57, 58], it has been shown that chronic cannabis users often display suppressed dopamine release [59–61]. Furthermore, striatal dopamine levels are also found to be reduced in patients with dual diagnoses, including schizophrenia patients with a history of cannabis use under controlled and stressed conditions [62, 63]. These observations would suggest that using a simple novelty-induced locomotion test to model positive symptoms in cannabis-treated rodents oversimplifies a complex relationship. Therefore, we suggest refined protocols to study positive symptoms are necessary. For example, experiments that performed a pharmacological challenge with psychostimulants such as amphetamine and cocaine revealed a significantly augmented difference in the locomotor activity between animals treated with CB1R agonists and controls [64–67]. However, our current study did not identify sufficient data from these experiments to conduct a quantitative analysis of the overall effects. Over the years, several other methods for assessing positive symptom-like behaviour have also been developed, such as models of “altered reality testing” through Pavlovian conditioning [68, 69], artificial manipulation of perceptual decision making [70, 71], and a more recent development which probes experimentally controlled auditory hallucinations in rodents [72]. Together, these improved behavioural paradigms hold great potential as more robust tools for modelling positive symptoms in rodents.

### CBD in schizophrenia

In this meta-analysis, we identified 9 publications that examined the effects of CBD on schizophrenia-like behaviour in wildtype animals. We found that chronic adolescent treatment of CBD was associated with a moderate but significant effect on improving fear memory recall in the fear conditioning task. However, results from other tests were non-significant and some likely underpowered.

While the effects of CBD alone might be less prominent, some preclinical studies show that CBD reduced hyperlocomotion induced by psychotomimetic agents like amphetamine and ketamine [73], as well as in glutamatergic dysfunction models of schizophrenia [74]. Social and sensorimotor deficits were also shown to be alleviated by chronic CBD treatment in MK801-induced schizophrenia models [74] and in stress-induced models [75, 76]. Along the same lines, it has been proposed that CBD may mitigate the psychotic effects of CB1R agonists by acting as a negative allosteric modulator of CB1R activity [77, 78], and/or modifying downstream signalling [79]. Here, we identified a need for more research to elucidate the impact of CBD and THC co-administration during adolescence, as existing evidence is limited and inconsistent.

### Sex-mediated effect of cannabinoids in rodents

Preclinical and clinical studies have consistently reported sex-dimorphic effects of cannabinoids [80, 81]. However, subgroup analyses from the current meta-analysis did not reveal sex as a significant mediator in 8 of 9 behavioural tests included. This result should be interpreted with caution, as the uneven data distribution and low statistical power of our subgroup analysis could explain the lack of sex-mediated effect. We also noticed that although sexually dependent effects were frequently observed among individual studies, the direction and magnitude often varied greatly, sometimes contradictory. Therefore, an average effect of these studies might not be informative. Herein, we echo the calls for more inclusion of female animals in preclinical research [82, 83], as this is essential for improving its clinical translation.

### Limitations and challenges

This meta-analysis provides a rigorous and comprehensive assessment of the effects of adolescent cannabinoid exposure on rodent behaviour. However, we must consider the limitations of our study. Firstly, the great disparity in the experimental settings of the animal studies, such as the age of exposure and the treatment protocols, could profoundly influence the animals’ response to drugs, which in turn increases heterogeneity and reduces the validity and generalizability of our findings. To circumvent this, we had initially planned additional subgroup analyses to explore the impact of dose and age of onset. However, such an effort was restricted due to the uneven distribution of data across subgroups, and no conclusive association could be observed. For reference data, see Supplementary Figs. S1 and S2.

Second, many studies did not report essential information on experimental design and outcome data adequately. This hindered the risk of bias assessment and sensitivity analysis, and the outcome data had to be frequently extracted using a digital ruler software from graphical representations. Third, we could not include three behavioural tests for schizophrenia-like behaviour in the quantitative synthesis, because they either had insufficient data reported, or they lacked standardised protocol and outcome measurement. Fourth, some of our subgroups included data from multiple experiments within the same study rather than from different studies. This could potentially impact the accuracy of the overall effect estimates due to uncontrolled study-specific factors. Finally, the publication bias observed in our meta-analyses may indicate selective reporting of significant data, which could potentially result in inflated or biased summary effects.

### Future directions

In conducting this meta-analysis, we found that the definition of adolescence in rodents varied greatly, with a window ranging from P23 to P45. Adolescence marks a critical period for neurodevelopmental changes, when the brain structure and function change immensely, and are highly susceptible to the effects of drugs [40, 84, 85]. Therefore, we highlight that standardising the adolescent period would improve comparability and interpretability. Another key challenge in studying the effects of cannabis on schizophrenia-like behaviour is to devise a drug delivery method that accurately reflects human cannabis use. Most of the current studies rely on the intraperitoneal route, which is convenient but may not capture the complexity and variability of human cannabis consumption patterns. We are encouraged by recent studies utilising more translational relevant models like inhaled cannabis vapour [86], but such studies remain uncommon. Moving forward, characterising cross-species pharmacodynamic/pharmacokinetic correlations for cannabinoids would enhance model validity and reliability. Investigating dose-response relationships and drug interactions would also inform model optimisation. Such efforts to improve translational relevance will accelerate insights into mechanisms linking human cannabis exposure and schizophrenia phenotypes, informing prevention and treatment.

While our meta-analysis indicates that chronic adolescent cannabinoid exposure in rodents leads to behavioural impairments relevant to schizophrenia, it is important to recognise that these impairments are not exclusive to schizophrenia-spectrum disorders. Cognitive and social deficits, for example, can also be present in other conditions such as depressive disorders [87] and autism-spectrum disorders [88]. Therefore, the behavioural tests used in this study, while indicative of schizophrenia-like symptoms, may also reflect broader psychopathological processes. Our careful phrasing of these symptoms as ‘schizophrenia-related’ or ‘schizophrenia-like’ underscores this broader implication but also reflects the aims of the studies included in the meta-analysis. Future research should aim to develop behavioural tasks that capture more specific effects of cannabinoid exposure across different psychopathological domains.

In conclusion, despite variation in experimental protocols and paradigms, the results of this meta-analysis confirm that chronic exposure to CB1R agonists during adolescence is associated with the expression of several schizophrenia-like behaviours in rodents. This supports findings from human epidemiological studies. Moving forward, standardisation of protocols, consideration of developmental periods and sex differences, and inclusion of diverse cannabinoid combinations will facilitate cross-species translation to elucidate the mechanisms linking adolescent cannabis use and schizophrenia phenotypes.

## Supplementary information

Supplementary Materials

Supplementary Data Collection Sheet

## Data Availability

The data supporting the findings of this study are available within this article and the Supplementary Data Sheet.
